# A quality improvement initiative for delayed umbilical cord clamping in very low-birthweight infants

**DOI:** 10.1186/s12887-016-0692-9

**Published:** 2016-09-13

**Authors:** Jeff Bolstridge, Tracy Bell, Barbara Dean, Amy Mackley, Gina Moore, Cheryl Swift, Dina Viscount, David A. Paul, Stephen A. Pearlman

**Affiliations:** Department of Neonatology, Christiana Care Health System, 4755 Ogletown Stanton Rd, Suite 217, MAP I, Newark, DE 19718 USA

**Keywords:** Delayed cord clamping, Neonatal blood transfusion

## Abstract

**Background:**

Due to clinical benefits, delayed cord clamping (DCC) is recommended in infants born before 37 weeks gestational age. The objective was to institute a delayed cord clamping program and to evaluate clinical outcomes one year after initiation.

**Methods:**

This study occured at Christiana Care Health System, a tertiary care facility with a 52 bed level 3 Neonatal Intensive Care Unit (NICU). A multidisciplinary team created a departmental policy, a DCC protocol and educational programs to support the development of a DCC program. A year after initiation of DCC, we evaluated two cohorts of very low birth weight (VLBW) infants (<1500 g) prior to (Cohort 1) and after initiation (Cohort 2) of DCC (*n* = 136 and *n* = 142 respectively). Chart review was conducted to evaluate demographic data and clinical outcomes. Analysis was completed with a retrospective, cohort analysis on an intention-to-treat basis.

**Results:**

There were no differences in demographic factors between the two cohorts. We demonstrated a 73 % compliance rate with the delayed cord clamping protocol and a decrease in the percentage of VLBW infants requiring red blood cell transfusion from 53.7 to 35.9 % (*p* = 0.003). We also found a decreased need for respiratory support in the second cohort with no increases in the balancing measures of admission hypothermia and jaundice requiring phototherapy. During the Control Phase ongoing monitoring and education has led to a 93.7 % compliance rate.

**Conclusions:**

A multidisciplinary team including key leadership from the obstetric and pediatric departments allowed for the rapid and safe implementation of DCC.

## Background

In recent years, the standard of care to immediately clamp the umbilical cord after delivery has been questioned. The practice of delayed cord clamping (DCC) for at least 30 s after the delivery of preterm infants has now fallen into favor at many medical centers in the United States [1, 2]. In December 2012, the American College of Obstetricians and Gynecologists (ACOG) and the American Academy of Pediatrics (AAP) released a joint statement supporting a 30 to 60 s delay in clamping the umbilical cord following the births of preterm infants. The single most important clinical benefit cited in the statement was a 50 % reduction in intraventricular hemorrhage (IVH) [3]. Additional reviews list other potential benefits including a decreased incidence of necrotizing enterocolitis, a reduction in transfusions for anemia [4–6] and a decreased need for respiratory support [7]. Though DCC has proven benefit, it has also been associated with an increased incidence of jaundice requiring phototherapy and delivery room hypothermia [5, 8, 9].

At our facility, delayed umbilical cord clamping was not being performed in preterm infants prior to the release of the ACOG/AAP statement. The implementation of delayed cord clamping required a coordinated, multi-disciplinary effort to educate physicians and staff involved in delivery room care. The aim of our quality improvement initiative was to successfully implement DCC for infants born preterm. One year after initiation of DCC, we retrospectively evaluated our compliance and clinical outcomes.

## Methods

This study received approval by the Institutional Review Board of Christiana Care Health System. All data were collected in a de-identified manner. This study in no way affected the routine medical care provided to infants at our Neonatal Intensive Care Unit (NICU).

Christiana Care Health System is a regional tertiary care teaching hospital located in Newark, Delaware with a 52 bed level 3 NICU. There are approximately 6500 deliveries and 1100 NICU admissions annually. At the time of this study there were 12 neonatologists and 55 obstetricians on staff in our institution.

After the release of the ACOG/AAP Committee Opinion in December 2012, a multi-disciplinary team was formed to review the recommendations and initiate a DCC program using quality improvement science. The team was comprised of NICU, Labor and Delivery and Perinatal nursing specialists, a NICU staff nurse, a neonatal research coordinator, a quality and safety data coordinator, a neonatologist, an obstetrician, a neonatal fellow, and a medical student. Working together with the leadership of the Obstetrics Department, the team developed a departmental policy on DCC. Furthermore, the team created individualized power point presentations for physicians and nursing staff to provide in-service education for nurses and grand rounds lectures for pediatric and obstetric providers. Committee members served as champions who addressed concerns of their own constituents. In addition, a detailed protocol for performing DCC was developed. Signage was displayed in each delivery room to ensure understanding of the proper technique.

We developed exclusion criteria for DCC based on local and national expertise. The exclusion criteria were: monochorionic-monoamniotic twins, any twin gestation with the second twin showing signs of distress, fetal asystole, complete abruptio placenta, anterior placenta, and any maternal condition requiring immediate attention, including maternal hypotension or cardiac arrest. Ultimately, it was the obstetrician’s decision to provide DCC in each delivery.

The multi-disciplinary committee developed a standardized DCC protocol, which was the same regardless of the mode of delivery. After the complete vaginal expulsion of the neonate or the extraction of the neonate from the uterus, our protocol called for delayed clamping of the umbilical cord for 60 s, which concurs with the 2012 ACOG/AAP recommendations of DCC for 30–60 s. During those 60 s, the neonate is wrapped in a warm, dry, sterile towel and for vaginal birth held 10 cm below the level of the introitus or, in the case of cesarean delivery, placed on the operating room table beside the mother. At any time, DCC could be stopped during a delivery at the discretion of the team managing the mother or baby. Apgar scores are reported from the time of delivery, not from the timing of cord clamping. A neonatal provider at the delivery verbally announced 15 s intervals and at 60 s the cord was clamped and cut and the infant was placed under the radiant warmer. In the early stages of the study there was a slight increase in the number of hypothermic patients. In response, the protocol was modified to sandwich the babies between 2 pre-warmed blankets during the 60 s of DCC in addition to the polyethylene wrap was used to improve thermoregulation once the baby was placed under the radiant warmer. Delivery rooms are maintained at 68 to 70° Fahrenheit. Following delivery and DCC, the NICU team provided care as needed. The routine care of the infant was in no way affected by our study and was in accordance with protocols already in existence at our facility. Resuscitation was performed utilizing the Neonatal Resuscitation Program guidelines published by the AAP [10].

Our initiative to provide delayed umbilical cord clamping began on July 1, 2013. Patients who qualified for DCC were all preterm (<37 weeks gestation) infants. However, for the ease of data collection and in order to focus on the highest risk population we only included inborn VLBW infants from July 1, 2012 to June 30, 2014 in our data analysis. This decision was predicated on the assumption that the population of VLBW infants is the one most likely to benefit from this intervention. We analyzed two cohorts; infants born before the DCC initiative from July 1, 2012 to June 30, 2013 (Cohort 1 – Pre-DCC) and infants born after the DCC initiative from July 1, 2013 to June 30, 2014 (Cohort 2 – Post-DCC). Our primary outcome measure was the effectiveness of our QI approach on the implementation of delayed cord clamping. With regards to the other clinical outcomes measured, we made an a priori decision to include all patients from Cohort 2 in the analysis, regardless if they received delayed cord clamping or not. In QI initiatives rarely is the entire population exposed to the intervention and by including all patients our data would be generalizable. Demographic information and clinical outcomes were obtained by thorough chart review. The data were recorded and saved on protected computers only available to the researchers. The demographic data included birthweight, gestational age, 1 and 5 min Apgar scores, and in-hospital mortality rates. Clinical outcomes collected included hypothermia upon NICU admission defined as less than 36 °C. Also packed red blood cell (pRBC) transfusion, respiratory support, phototherapy, early and late onset sepsis, IVH, periventricular leukomalacia, and necrotizing enterocolitis were tracked throughout the hospital stay. Sepsis was defined as a positive blood culture of a pathogenic organism. IVH is a known complication of preterm delivery [11] and was graded according to the system of Papile et al. [12]. Severe IVH was defined as Grades 3 and 4. Periventricular leukomalacia was defined as necrosis of the periventricular white matter as noted on ultrasound [13]. Necrotizing enterocolitis was defined as radiographic findings of gaseous distension of the bowel with pneumatosis or pneumoperitoneum along with associated clinical findings of abdominal distension and feeding intolerance [14]. Balancing measures included use of phototherapy, as a marker for hyperbilirubinemia, and the incidence of hypothermia. The decision to transfuse was made clinically at the discretion of the attending neonatologist, in accordance with our unit’s recommendations based on the Premature Infants in Need of Transfusion (PINT) study [15]. This transfusion policy did not change during the study period.

During the control phase of this initiative, we continue to monitor DCC compliance with monthly reports.

Continuous variables were analyzed by Student’s *t*-test and categorical data were compared using Chi square analysis. STATISTICA was used in data analysis. The manuscript was prepared using the Standards for Quality Improvement Reporting Excellence (SQUIRE 2.0) Guidelines [16].

## Results

**Fig. 1:**
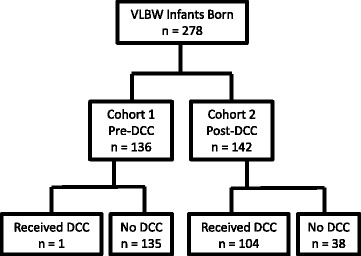
Patient Inclusion

There were 136 infants in cohort 1 (pre-DCC) and 142 infants in cohort 2 (post-DCC). Fig. 1 outlines the patients involved in the study. The percentage of babies who received delayed cord clamping is 0.7 % in Cohort 1 and 73.0 % in Cohort 2 (*p* = 0.0001). Table 1 shows the demographic data. There were no differences in gestational age, multiple gestations, birth weight, mortality rate, or 1 and 5 min Apgar scores between the two cohorts. Table 2 shows clinical outcomes. There was a decrease in the percentage of infants requiring packed red blood cell transfusion at any time during the hospital stay from 53.7 to 35.9 % (*p* = 0.003). There were decreases in the use of CPAP and ventilatory support throughout the hospital stay. There were reductions in delivery room intubation and delivery room chest compressions following the institution of DCC. Cohort 2 also had a decrease in the incidence of late-onset sepsis. There were no differences between cohorts in the incidence of IVH of any grade, severe IVH, periventricular leukomalacia, early-onset sepsis, necrotizing enterocolitis, or use of inotropic support during the hospitalization. Importantly, there were no increases in the balancing measures of admission hypothermia or the need for phototherapy.

**Table 1:** Demographic data

	Cohort 1 (*n* = 136)	Cohort 2 (*n* = 142)	*P*
Gestational age (weeks)	27.8 ± 3.0	28.3 ± 2.8	0.17
Birth weight (grams)	1010 ± 297	1050 ± 306	0.27
Multiple gestation	34 (25.0 %)	24 (16.9 %)	0.10
Received delayed cord clamping	1 (0.7 %)	104 (73.2 %)	<0.001
Mortality	15 (11.0 %)	12 (8.5 %)	0.47
1 min Apgar score less than 5	66 (48.5 %)	56 (39.4 %)	0.13
5 min Apgar score less than 5	18 (13.2 %)	15 (10.6 %)	0.49

**Table 2:** Clinical outcomes

	Cohort 1 (*n* = 136)	Cohort 2 (*n* = 142)	*P*
Delivery room intubation	78 (57.4 %)	55 (38.7 %)	0.002
Delivery room chest compressions	24 (16.6 %)	9 (6.3 %)	0.004
Delivery room normal saline bolus	1 (0.7 %)	4 (2.8 %)	0.19
Delivery room epinephrine	4 (2.9 %)	4 (2.8 %)	0.95
Normal saline bolus given on first day of life	11 (8.1 %)	6 (4.2 %)	0.18
Transfused PRBC	73 (53.7 %)	51 (35.9 %)	0.003
Hypothermia upon NICU admission	40 (29.4 %)	42 (29.6 %)	0.97
Phototherapy	116 (85.3 %)	124 (87.3 %)	0.62
Ventilation at any time during hospitalization	94 (69.1 %)	78 (54.9 %)	0.015
CPAP at any time during hospitalization	114 (83.8 %)	102 (71.8 %)	0.016
Inotropic support	23 (16.9 %)	17 (12.0 %)	0.24
Intraventricular hemorrhage, any grade	34 (25.0 %)	35 (24.6 %)	0.95
Severe intraventricular hemorrhage	18 (13.2 %)	10 (7.0 %)	0.086
Periventricular leukomalacia	2 (1.5 %)	6 (4.2 %)	0.17
Patent ductus arteriosus	51 (37.5 %)	42 (29.6 %)	0.16
Early onset sepsis	2 (1.5 %)	2 (1.4 %)	0.96
Late onset sepsis	14 (10.3 %)	5 (3.5 %)	0.025
Necrotizing enterocolitis	4 (2.9 %)	11 (7.7 %)	0.076

The implementation of delayed umbilical cord clamping was subjectively well received by most physicians and staff as evidenced by the early adoption of this new protocol. Through ongoing monitoring of the process we were able to detect when there was provider resistance to DCC. When this was noted, additional education and support was offered to providers as needed. Efforts to maintain compliance with DCC continue during the control phase. Our compliance with our delayed cord clamping policy is currently at 93.7 % in all infants less than 37 weeks gestation.

## Discussion

In our single center study, the implementation of delayed umbilical cord clamping was successful, as evidenced by the initial 73 % compliance rate of VLBW infants in the year following the initiative. There were important associated clinical benefits including decreases in pRBC transfusions, decreased delivery room intubations and chest compressions, decreased respiratory support and late onset sepsis. There was no concomitant rise in balancing measures such as admission hypothermia and the need for phototherapy.

The implementation of DCC led to a reduction in the number of blood transfusions given to VLBW infants at our facility. Neonatal blood transfusion carries many risks, including fluid overload, necrotizing enterocolitis, and transmission of blood-borne pathogens [17]. DCC has been associated with a reduction in the need for blood transfusion [2, 4, 6, 7, 18, 19] and increases in hematocrit values [5–7, 18–20]. Much literature has been devoted to quantifying the transfusion that occurs in the moments following birth. Farrar et al. weighed term infants after birth with the cord intact, showing that an approximate 25 ml/kg placental transfusion occurs [21]. The majority of the placental transfusion occurs in the first 15 to 60 s after delivery [1, 6]. Studies in preterm lambs show that this placental transfusion has many physiologic benefits, including less variation in heart rate and carotid artery pressure [22]. In the preterm population that we studied, DCC reduces the need for transfusion acutely during their initial hospitalization. In preterm and term infants, DCC decreases the need for late transfusions by reducing the incidence of iron deficiency anemia. Though improvements in hemoglobin concentrations are more transient, patients receiving early cord clamping have reduced iron stores and are twice as likely to be iron deficient at three to six months of age [5].

Our study found a decrease in delivery room intubation, as well as ventilation and CPAP at any time during hospitalization of VLBW in the DCC cohort. Song et al. found decreases in respiratory support for very preterm infants receiving 60 to 75 s of placental transfusion [7]. The decrease in intubations could have been explained by a concomitant effort in our facility to reduce bronchopulmonary dysplasia (BPD), though this would not explain the decrease in CPAP use. It is possible that DCC gives the infant time to establish ventilation and lung volume prior to resuscitation.

We examined delivery room interventions and found decreases in the number of infants requiring delivery room intubation and chest compressions without any concomitant increase in balancing measures including mortality and low Apgar scores. In October 2015, the American Heart Association and the AAP released new guidelines for cardiopulmonary resuscitation of the neonate. The guidelines recommend DCC for 30 to 60 s for most vigorous term and preterm infants, though they state there is insufficient evidence to recommend DCC for infants requiring resuscitation [23]. Future work should include possible methods to provide resuscitation to the neonate during DCC.

DCC has also been shown to have some negative effects. Cochrane reviews have found increases in peak bilirubin [4] and phototherapy [5] to be associated with DCC. Concerns have also been raised for an increased risk of hypothermia. We found no increase in utilization of phototherapy or in the incidence of hypothermia. Interestingly, some studies have shown DCC to be associated with less admission hypothermia [7, 18, 20]. Umbilical cord milking could serve as an alternative to DCC as it may be faster and hence reduce the risk of hypothermia or delayed resuscitation [24, 25].

In this study, we detail the steps taken to initiate DCC from a single-center perspective utilizing a quality improvement approach. The high initial compliance rate shows that by employing a multidisciplinary team and by using QI methodology, DCC can be successfully and safely implemented. The results from our study only examine the outcomes of VLBW infants who often require the most aggressive delivery room management and yet we still demonstrated a high compliance rate for DCC. We did not specifically address the value of this intervention in larger preterm and term infants but previous studies have done so.

The major limitation of this study is its low statistical power to examine rare outcome events such as NEC or PVL. As with any retrospective analysis the results are not necessarily attributable to DCC but may be secondary to other clinical changes. During this study, our facility had a concomitant effort to reduce BPD by decreasing ventilation performed, though this would not explain the decrease in the need for CPAP and chest compressions.

## Conclusion

Delayed umbilical cord clamping has a growing body of research showing many clinical benefits. Implementation of DCC can be a challenging undertaking but through the use of a quality improvement approach our efforts were successful achieving a rapid high compliance rate for DCC. We evaluated VLBW infants, who often are those patients who require additional resuscitation and found decreases in delivery room and respiratory interventions performed with no increase in negative balancing measures. Delayed cord clamping can be successfully and safely implemented using quality improvement methodology and by engaging a multidisciplinary team.

## Abbreviations

AAP: American Academy of Pediatrics
ACOG: American College of Obstetricians and Gynecologists
BPD: Bronchopulmonary dysplasia
DCC: Delayed cord clamping
IVH: Intraventricular hemorrhage
NICU: Neonatal intensive care unit
pRBC: Packed red blood cells
VLBW: Very low birth weight
